# Highly hard yet toughened *bcc*-W coating by doping unexpectedly low B content

**DOI:** 10.1038/s41598-017-09807-9

**Published:** 2017-08-24

**Authors:** Lina Yang, Kan Zhang, Mao Wen, Zhipeng Hou, Chen Gong, Xucheng Liu, Chaoquan Hu, Xiaoqiang Cui, Weitao Zheng

**Affiliations:** 1 State Key Laboratory of Superhard Materials, Department of Materials Science, Key Laboratory of Automobile Materials, MOE, and Jilin University, Changchun, 130012 People’s Republic of China; 2 King Abdullah University of Science and Technology (KAUST), Physical Science and Engineering (PSE), Thuwal, 23955-6900 Saudi Arabia

## Abstract

Either hardness or toughness has been the core interest in scientific exploration and technological pursuit for a long time. However, it is still a big challenge to enhance the hardness and toughness at the same time, since the improvement of one side is always at the expense of the other one. Here, we have succeeded in dealing with this pair of conflict based on tungsten (W) coating by doping boron (B) via magnetron co-sputtering. The results reveal that the introduction of low concentrations of B (6.3 at. %), in the doping regime, leads to the formation of W(B) supersaturated solid solution with refined grains. Meanwhile, the doping-induced higher compressive stress, higher H/E^*^ and denser microstructure result in a surprising combination of improved hardness (2 × larger than pure W) and superior toughness (higher crack formation threshold compared to pure W). We believe this is an innovative sight to design new generation of transition-metal-based multifunctional coatings. Besides, our results are applicable for industrial application because it can be realized by simple manufacturing approaches, e.g. magnetron sputtering technology.

## Introduction

Hardness and toughness seem as a pair of conflicts^1^: the improvement of one side is at the expense of the other one. Nevertheless, it is of great importance for engineering application if a material exhibits both high hardness and toughness. Consequently, the work associated with simultaneously increasing these two properties is filled with challenge but significance. To date, this task has been well accomplished by embedding carbon nanotubes (CNTs) into brittle ceramics like silicon carbide^2–6^ and alumina^7–9^. In this way, as-synthesized composite materials exhibited both improved hardness and toughness, wherein the intrinsic attributes of CNTs dominates the strengthening and toughening effect. Besides, rare data for simultaneous investigation in terms of hardness and toughness has been available on alloying elements, instead, most efforts have been focused on improving the single aspect, either hardness or toughness. Just as the reports about the metal tungsten (W), which is not as hard as ceramics but exhibits brittle behavior^10, 11^. To improve its toughness, enormous attentions have been put on alloying W with transition metals like rhenium (Re)^12, 13^, iridium^14^, vanadium^15^ and tantalum^16^, etc. In which, Re attracts much attention since the tough W can be obtained on account of “Re ductilizing effect”^17^, this theory is based on optimized dislocation-core structure, along with lower Peierls stress. Nevertheless, its application is subject to a certain limitation when taking account of cost. In addition to the toughness enhancement, another important subject for W is to improve its hardness. Aiming at this point, some researchers employed the same method of alloying with other transition metals like Lu^18^ and V^19^. However, the strengthening efficiency is limited because the substitutional atoms can only cause slight lattice distortion and weak solid solution strengthening.

Recently, limited reports concerning metal films doped by few small atoms attract much attention. For instance, just incorporation of a small concentrations of B (5 at. %) by magnetron radio frequency sputtering into Cr film to form solid solute Cr(B) film, 2.6× increase in hardness can be obtained^20^. Similarly, Shang *et al*.^21^ have employed the same method to deposit Al film alloyed by 1.89 at. % B, and found that the film exhibited nearly 2-fold microhardness relative to pure Al. Besides, more phenomena relevant to the strengthening effect of suchlike alloying small atoms into metallic films could be traced to refs 22 and 23. Nearly all researchers attribute the attained hardening to fine grains and solid solution strengthening, which is supported by the fact that the interstitial atoms can cause more severe lattice distortion of matrix material in comparison with substitutional atoms. What is worth mentioning, in 2016, L. Hultman group^23^ have presented a beautiful work, they first reported the N-doped *bcc*-Cr films exhibiting the unique combination of high hardness and excellent toughness at low N concentrations, ~5 at. %. Particularly, the film was synthesized in virtue of specific high-power pulsed magnetron sputtering (HIPIMS)^24^. Through this deposited technology, synchronized Cr-ions rather than gas-ions irradiation can be monitored and predominantly selected to arrive at the growth surface, thus dense and atomically-smooth film with less lattice defect can be prepared. Inspired by the works above, for the transition metal W, two questions remain: (1) whether the hardness could be enhanced by addition of unexpectedly low level B content just through simple magnetron co-sputtering method? (2) As W is much brittle than Cr, could the small quantity of B atoms also improve the toughness?

Taking account of above questions, we deposited low-concentration B doped *bcc*-W (α-W(B)) coating via magnetron co-sputtering technology. Besides, pure W and W_2_B were prepared as references for comparison. Our studies revealed that the W film reinforced by 6.3 at. % B showed promising results: in comparison with pure W, 2-fold increase in hardness and drastically enhanced toughness can be achieved. Moreover, it is worth mentioning that such foreign-element doping is an effective approach not only for enhancing mechanical properties, but also for improving electrical^25, 26^, optical^27, 28^ and magnetic features^29, 30^ of materials, which are equally important for high technical products. Hence, our findings possess a certain scientific and applied values, and could help develop a new group of transition-metal-based multifunctional coatings.

## Results and Discussion

### Composition, chemical bonding and structure

**Figure 1:**
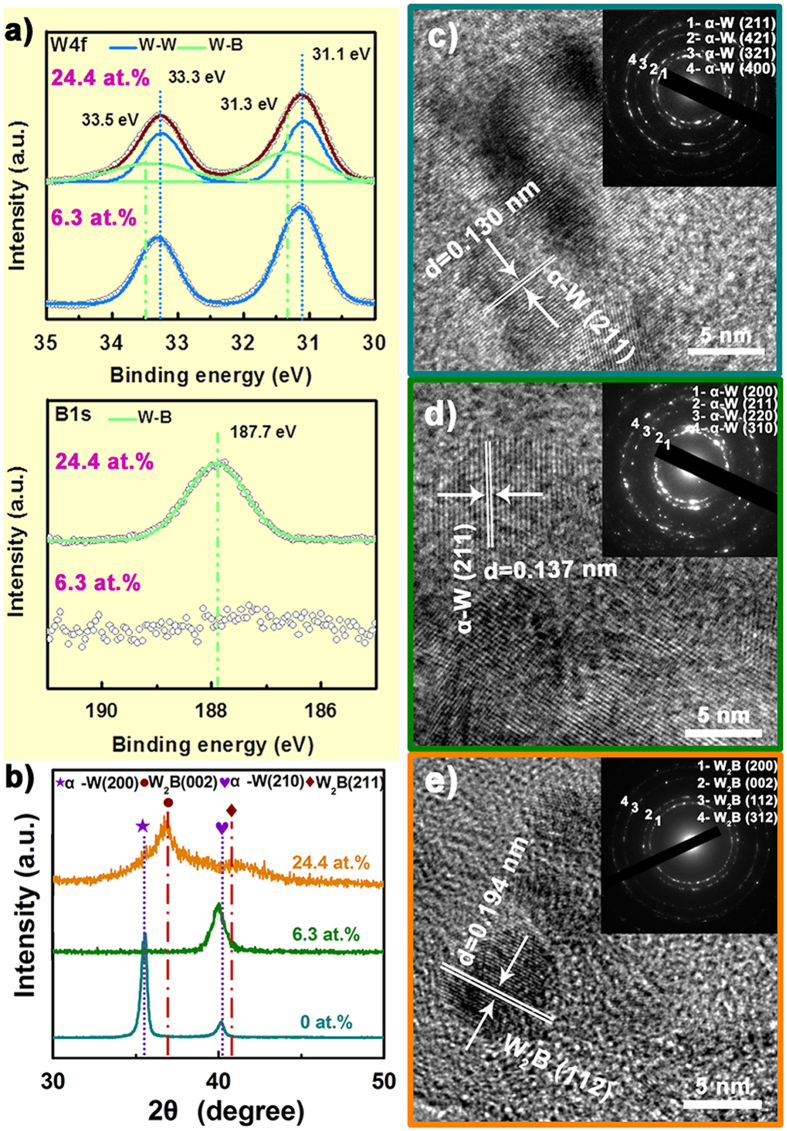
(**a**) XPS W4f and B1s spectra for the coating with B content of 6.3, and 24.4 at. %. (**b**) XRD patterns for all coatings as a function of B content. (**c**–**e**) HRTEM and corresponding SAED images (insets) formed at the coating with B content of 0 at. % (**c**), 6.3 at. % (**d**), 24.4 at. % (**e**).

According to pre-etching XPS results, we obtained 0, 6.3 and 24.4 at. % B content by regulating B target power to 0, 70 and 400 W, while W target power was kept constant at 70 W (Table 1). To further explore the chemical bonding structure between W and B, two typical core-level spectra in W4f and B1s energy regions are shown in Fig. 1a. In the W4f spectrum, the apparent double peaks for W4f_7/2_ and W4f_5/2_ can be observed, by means of Gaussian functions, they were deconvoluted into four peaks. Wherein two peaks located at 33.3 eV and 31.1 eV are considered as metallic W-W bonds^31^, while two other peaks centered at 33.5 and 31.5 eV can be assigned to W-B bonds, since a shift to high-energy side occurs when charges transfer from W to B. We note that only W-W bonds but no W-B bonds exist in the 6.3 at. % B-containing coating, and it can be further confirmed by the high noise level of the corresponding B1s spectrum, implying that all the B atoms occupy the interstitial sites of W lattice. When B content is 24.4 at. %, B1s spectrum exhibits a symmetrical binding energy peak at 187.7 eV, indicating that the single chemical state exists between W and B atoms, which is attributed to W-B bonds, while no B-B bonds exist in the film. Moreover, the 24.4 at. % B atoms in the coating can only to form sub-stoichiometric W_2_B phase, so it can be concluded that no interstitial B atoms exists in the coating with 24.4 at. % B.

**Table 1:** Experimental parameters, composition, grain sizes (D) and H/E^*^ for all coatings.

As-deposited coating	W target power (W)	B target power (W)	Composition (at. %)		D (nm)	H/E*
			W	B		
W	70	0	100	0	15.3	0.066 ± 0.01
α-W(B)		70	93.7	6.3	8.6	0.102 ± 0.01
W_2_B		400	75.6	24.4	3.1	0.124 ± 0.02

Figure 1b exhibits the XRD pattern as a function of B content. For pure W, two sharp peaks at 2*θ* = 35.62° and 40.16° are related to (200) and (210) plane of body-centered cubic α-W (JCPDF:65-6453), respectively. With B content increasing to 6.3 at. %, α-W (200) peak disappears and only α-W (210) remains. Combined with its XPS result, we conclude that the single peak should be assigned to B doped α-W (α-W(B)), and the solute B atoms expand α-W lattice to make it shift towards lower Bragg-angle relative to the standard diffraction peak of α-W (210). It’s worth noting that this coating comprises supersaturated solid solution of W, as the solute B content (6.3 at. %) is far larger than the equilibrium solid solubility limit of W solid solution (<1 at. % B^32^). The formation of such supersaturated solid solution is due to the non-equilibrium characteristic of magnetron sputtering^21^. As B content increases to 24.4 at. %, the XRD pattern gives two new broad peaks at 2*θ* = 37.52° and 40.61°, which can be identified as (002) and (211) reflections of tetragonal γ-W_2_B (JCPDF: 25-0990), respectively, showing a good consistency with the XPS result. Notably, as-deposited W_2_B coating possesses extremely poor crystallinity. Here, grain sizes calculated roughly by Debye-Scherrer’s formula^33^ are depicted in Table 1. It is found that the average grain size decreases with increasing B content, from 15.3 nm for pure W to 8.6 nm for α-W(B), and decrease to 3.2 nm as the W_2_B coating was obtained.

To acquire more information about microstructure, we employed TEM analysis, as displayed in Fig. 1(c–e). In Fig. 1c, the pure W shows a large-area α-W (211) with interplanar spacing (*d*) of 0.130 nm from the HRTEM image, and the SAED pattern also confirm that the sample consists of α-W phase. When B content is 6.3 at. %, the inset still shows a typical α-W SAED pattern, without any sign of B or borides phase, meanwhile, the grain size identified by HRTEM image is around 10 nm, consistent with XRD result that the grain size is 8.6 nm. In contrast, the *d*_211_ increases to 0.137 nm for the film with 6.3 at. % B, in agreement with the peak shift toward low angle in XRD, proving that a certain lattice expansion occurs due to the interstitial sites of W lattice are partially occupied by B atoms. As B content increases to 24.4 at. %, the diffraction ring (Fig. 1e) correspond to the pure W_2_B phase, but large-area amorphous phase appears in the HRTEM observation, which supports the XRD analysis. Notably, the *d* value of W_2_B (112) is 0.194 nm, slightly lower than the theoretical value at 0.204 nm (JCPDF: 25-0990). This is ascribed to the presence of large amount of B vacancies in W_2_B phase due to B deficiency.

### Growth and morphology

**Figure 2:**
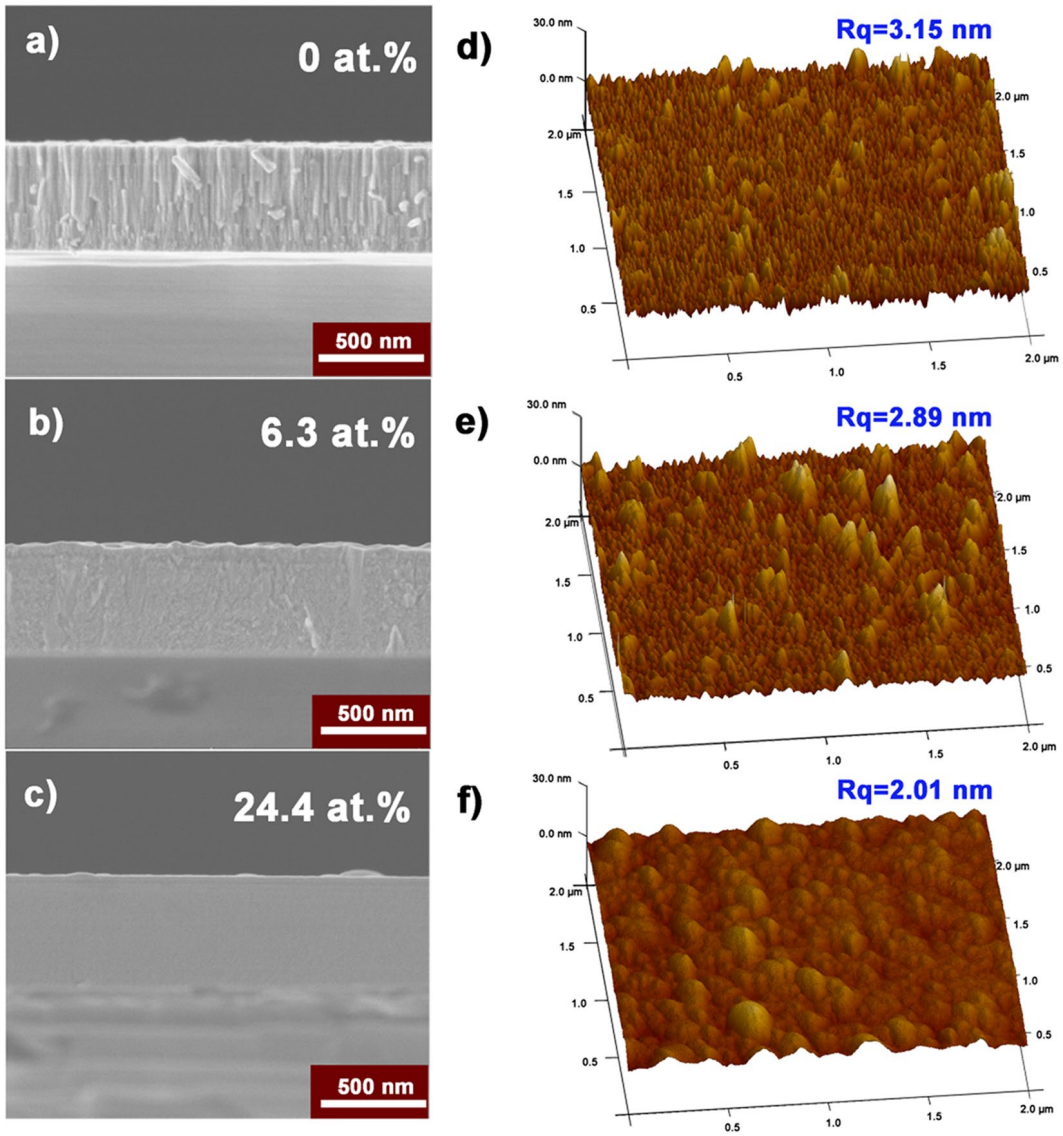
(**a**–**c**) Cross-sectional SEM images for the coatings with B content of 0 at. % (**a**), 6.3 at. % (**b**), 24.4 at. % (**c**). (**d–f**) AFM 3D micrographs in dimensions of 2 × 2 μm^2^, the root-mean-square roughness (Rq) is marked.

To detect the effect of B doping on the coating growth, the cross-sectional SEM was performed on all samples, as can be seen from Fig. 2(a–c). For the pure W coating (Fig. 2a), the fine structure with ~20-nm diameter columns is revealed, which is typical for metallic films. But such structure is almost invisible in the coating with 6.3 at. % B (Fig. 2b), so that the cross-section observation shows nearly featureless except for some shallow dimples. With B content increasing to 24.4 at. % (Fig. 2c), the coating exhibits no-columnar but flat cross-section. Obviously, the addition of boron atoms into the W coating influences the microstructure evolution and results in the microstructure altering from columnar to featureless, accompanied with a denser microstructure. To understand if such columnar-featureless microstructure evolution correlates with the morphology of growing layers, atomic force microscopy (AFM) is carried out on each coating. The surface three-dimensional images of coatings with various B content are presented in Fig. 2(d–e), and the root-mean-square roughness (Rq) is also marked. It is found that the pure W possesses rather deep groove, which originates from the columnar structure, thereby the largest Rq of 3.15 nm can be obtained. When adding B atoms to pure W, the protuberances get less sharp, and Rq reduces slightly to 2.89 and 2.01 for α-W(B) and W_2_B, respectively. The deduced grain size (Table 1) may be responsible for such variation. Note that all coatings exhibit Rq values lower than 5 nm, thus relatively high-quality coatings were prepared.

### Effect of B doping on the hardness

**Figure 3:**
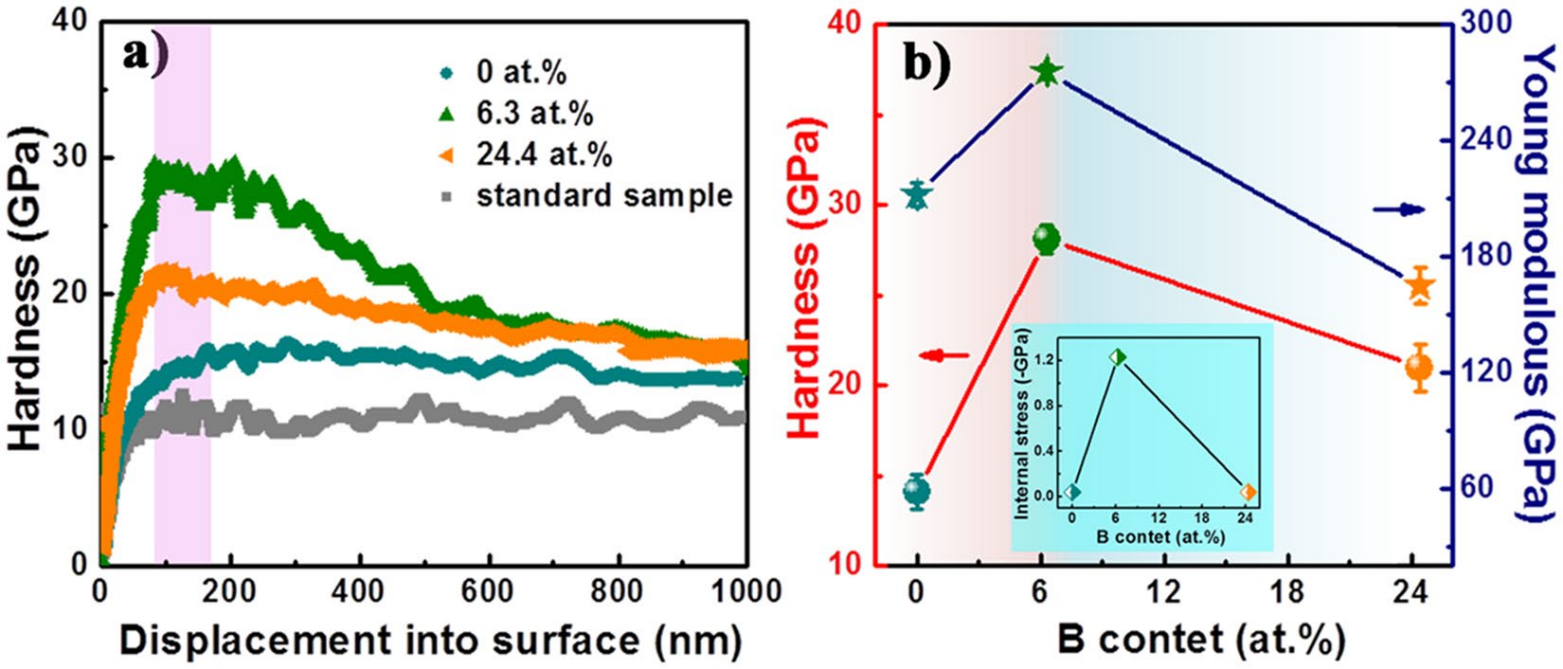
Hardness-displacement curves for the fused silica standard sample and as-deposited coatings (**a**), hardness and Young modulous for the coatings as a function of B content (**b**), the internal stress data is added to inset (**b**).

To get reliable hardness, the calibration for fused silica standard sample was performed. As shown in Fig. 3a, the standard sample exhibits a steadily standard value at about 10 GPa as indentation depth increases, while the hardness for all specimens increases first then decreases gradually because as-deposited coatings tend to be affected by surface roughness and substrate. Hence, it is acceptable to take the hardness value at around 1/10 of impression depth to avoid the effects of surface and substrate. Figure 3b plots the hardness and elastic modulus for coatings with different B content. As B content increases, the hardness increases drastically and decreases afterwards. For pure W, the hardness is 14.1 ± 0.93 GPa, and the maximum value of 28.1 ± 0.79 GPa can be obtained in α-W(B) coating, it is far larger than pure W_2_B at 20.1 ± 1.29 GPa. The elastic modulus with B content follows the hardness to show a similar tendency.

**Figure 4:**
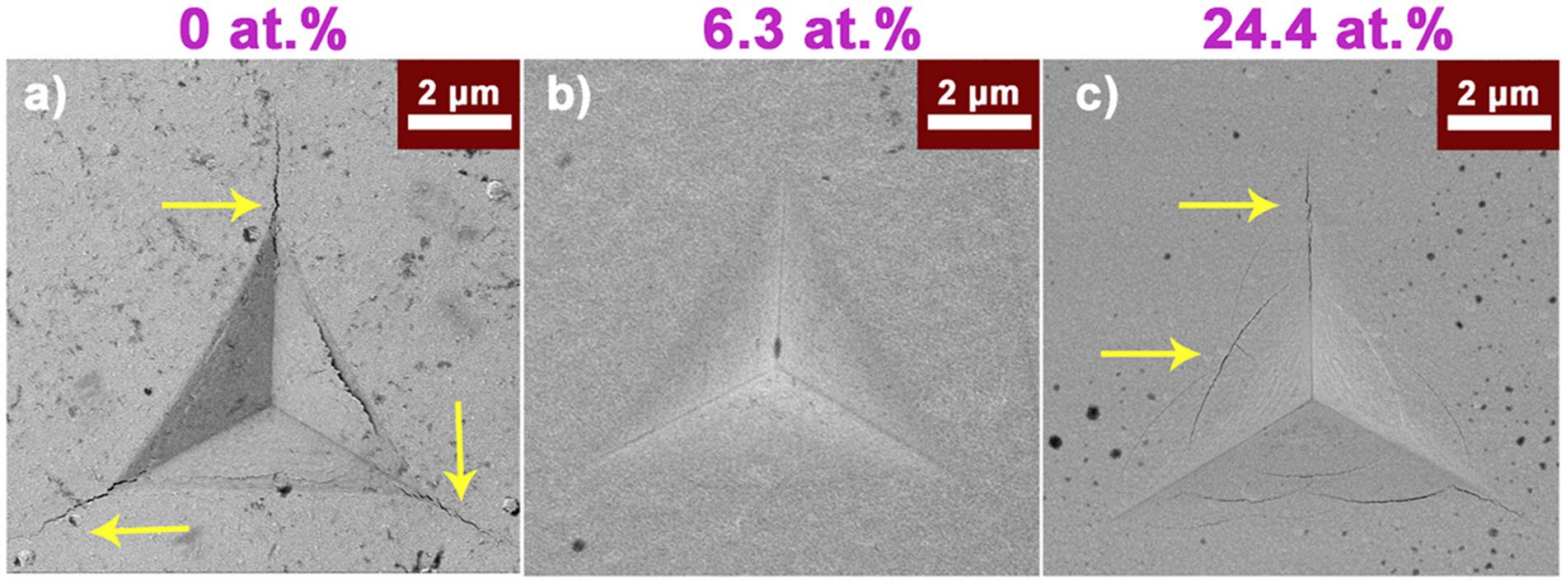
SEM images of the indentations developed on the coatings with B content of 0 at. % (**a**), 6.3 at. % (**b**), 24.4 at. % (**c**).

As we known, internal stress (IS) is an important factor affecting the hardness of coating, it was calculated and displayed in the insert of Fig. 4b. The result shows that the IS is compressive stress in nature, and the IS of pure W, α-W(B) and W_2_B coatings is 0.04 ± 0.01, 1.23 ± 0.06 and 0.04 ± 0.04 GPa, respectively, following the same trend as the hardness. Based on d’Heurle’s model^34^, we know the bombardment effects of incident atoms or ions induce the generation of compressive stress during the film growing process. For pure W, the minimum stress at 0.40 ± 0.01 GPa can be obtained, as the ordered lattice can be arranged by only W atoms or ions, in this case, almost no defects or atoms displacement appear. As B content increases to 6.3 at. %, the α-W(B) coating exhibits the highest compressive stress at 1.23 ± 0.06 GPa, because all B atoms implant into the subsurface of film to occupy the interstitial positions of W, resulting in significant lattice expansion. For the W_2_B coating, a dropped stress value to 0.04 ± 0.04 GPa can be ascribed to the poor crystallinity caused by phase transformation from α-W(B) to W_2_B. Here, the compressive stress may provide partial contribution to the variation in hardness^35^. In comparison with pure W, the α-W(B) coating exhibits 2-fold increase in hardness. Based on above analysis of grain size, microstructure, and coating growth, it is rational to conclude that such high hardness is caused by the synergistic effect of finer grains of ~8.6 nm (fine-grain strengthening), supersaturated solid solution (solid solution hardening^36^), non-columnar growth and higher residual compressive stress. As B content further increases to 24.4 at. %, a dropped hardness to 20.1 ± 1.29 GPa can be obtained. And the intrinsically low hardness (lower than 20 GPa) estimated by Xiao *et al*.^37^ together with its defective lattice structure govern the lower hardness, although similar non-columnar features can be observed.

### Effect of B doping on the toughness

In order to investigate the role of B on the toughness, nanoindentation and SEM have been performed on the coatings with different B content. The SEM images of the indentations for all samples are demonstrated in Fig. 4. It is found that pure W reference sample (Fig. 4a) exhibits severe radial crack, according with its intrinsically poor toughness, just as the yellow arrows illustrated. Surprisingly, a perfect impression without any radial crack is present in the coating with 6.3 at. % B (Fig. 4b), meaning that incorporation of solute B atoms enhances the metallic W toughness. However, the W_2_B coating with 24.4 at. % B (Fig. 4c) shows pronounced circumferential cracks and few radial cracks, that is different fracture mode occurs^38^.

As far as we know, the cracks can only nucleate when the stress at the tip of pre-existing flaw beyond crack formation stress of the material, the qualitative comparation can refer to following equation^39^:

1 $$\frac{{\sigma }_{{\rm{tip}}}}{{\sigma }_{applied}}=1+2\sqrt{\frac{a}{\rho }}$$

wherein 2*a* and *ρ* is the length of crack and tip radius, respectively, the ratio of σ_tip_/σ_applied_ denotes stress concentration factor, serving as an indication of crack formation threshold, and it can be drastically decreased through decreasing grain size or flaw size to nanometer dimensions, since the crack size is proportional to grain size. Hence, crack initiation requires higher applied stress (σ_applied_) if the grain size reduces^40^, that is the crack initiation becomes difficult. This concept may work in this work, wherein the mean grain size of α-W(B) coating is 8.6 nm, only half as many as that of W (~15.3 nm). Comparatively speaking, larger grains make pure W coating prefers to form cracks when applied the same load (~200 mN), besides, the representative columnar structure (Fig. 2a) facilitates the crack propagation. Thereby, higher crack formation threshold driven by finer grains exists in the α-W(B) coating. And the refined grains may produce more complicated grain boundary and induce the crack bending or branching, in parallel, more energy will be consumed to allow cracks to propagate. For the W_2_B coating, abundant circumferential cracks and few radial cracks appear, despite ultrafine grains (~3.1 nm) and denser microstructure are obtained, which is because that the formation of cracks is a complex process relating to not only the grain size and microstructure, but also other factors like mechanical properties (H, E*) and internal stress^41^, and the cracks result from a combined action of all factors.

It has been well established that the H/E* ratio is an important factor for evaluating the crack resistance of coatings, and higher H/E* (>0.1) could prevent radial cracks^42–45^. To investigate the different fracture mode between pure W and W_2_B coating, we further calculated the H/E* (E* = E/(1−υ^2^), υ = 0.25), as summarized in Table 1. It is found that the ratio increases from 0.066 ± 0.01, 0.102 ± 0.01 to 0.124 ± 0.02 with B content increasing from 0, 6.3 to 24.4 at. %, thus both α-W(B) and W_2_B coating, relative to pure W, tend to prevent the formation of radial cracks, since the two samples obtain similar H/E* value and larger than 0.1. Together with their denser microstructure (Fig. 2b,c), the radial cracks are suppressed. More importantly, the higher compressive stress (1.23 ± 0.06 GPa) in α-W(B) coating also contributes to suppress the formation of radial cracks^41^. Hence, the α-W(B) coating keep perfect indention without any cracks, but abundant circumferential cracks arise in the W_2_B coating. As shown in the XRD and HRTEM of Fig. (1b,e), the poor crystallinity combines with the emergence of amorphous in the W_2_B coating may be the reason for the existence of the circumferential cracks, just as reported that the amorphous film tends to create circular cracks^41^.

## Conclusion

Here, we successfully prepared *bcc*-W coating doped by 6.3 at. % B (α-W(B)) via magnetron co-sputtering W and B targets. Besides, the other coatings including W, and W_2_B were synthesized further for comparison. The combined experiments of XPS, XRD and TEM confirmed the existence of α-W(B) single phase, and revealed its supersaturated solid solution feature. The addition of B atoms refines the W grains, decreasing the average grain size from 15.3 nm for pure W to 8.6 nm for α-W(B), and leads to higher compressive stress. Accordingly, the coating microstructure changes from columnar to featureless, along with relatively smooth surface. Based on the nanoindentation and SEM measurement, our studies reveal promising results: the incorporation of B in W film could remarkably improve its hardness from 14.1 ± 0.93 to 28.1 ± 0.79 GPa (2-fold increment than pure W), the synergetic contribution of fine-grain strengthening, solid solution hardening, denser microstructure and higher compressive stress governs the strengthening mechanism. The W_2_B coating possesses intrinsically low hardness (~20 GPa). Simultaneously, in comparison with pure W and W_2_B, the α-W(B) coating exhibits dramatically enhanced toughness (perfect impression without any crack), which can be mainly attributed to the higher crack formation threshold induced by finer grains, and the higher H/E* (>0.1) improving crack resistance. To sum up, such integrate of higher hardness and superior toughness could be realized by amazingly small (6.3 at. %) addition of B to W.

## Methods

Deposition of α-W(B) with a thickness of around 700 nm was conducted on (100) oriented Si wafers by magnetron co-sputtering W (99.95%) and B (99.95%) targets in Ar. Prior to deposition, the substrates were successively ultrasonic cleaned in acetone, alcohol, and deionized water for 20 min respectively, and then mounted at 8 cm from targets. The background pressure was pumped down to 1.0 × 10^−4^ Pa, then the working pressure was controlled at 0.8 Pa with Ar flow rate of 32 sccm. During deposition, to tailor different B content in coatings, the radio frequency power inserted B target ranged in 0–400 W, while the direct current power linked to W target was kept constant at 70 W. The substrate bias, temperature and rotated speed was fixed at −60 V, 200 °C and 10 rpm, respectively.

The thickness and radius curvature of coatings were measured by a surface profiler (AMBIOS XP-2). Particularly, the reliable data about curvatures of all samples were got by averaging the minimum value of each eight measurements along two orthogonal surface directions, and outlier data points were rejected, subsequently, the total residual stress was calculated using the Stoney equation^46^. The composition and chemical structure in all coatings were investigated by X-ray photoelectron spectroscopy (XPS, Perkin-Elmer PHI-5702), where sputtering cleaned by Ar^+^ for 500 s was carried out prior to XPS test. X-ray diffractometer (XRD, D8-tools) in θ–2θ configuration together with transmission electron microscopy (TEM, field emission JEOL 2010F) operated at 200 kV were employed to study their phase structures. The samples on the Cu grids for TEM analysis were spread with pieces of film peeled off from the surface slightly. To explore the growth condition of films when alloyed by B atoms, cross-section scanning electron microscope (SEM, SU8010) under 1-kV accelerate voltage and 8-cm working distance was carried out. Subsequently, the surface morphology was characterized by atomic force microscope using ScanAsyst mode in air (AFM, Dimension Icon, Veeco Instruments/Bruker, Germany). To determine the hardness for all samples, we introduced nanometer-scale indents (nanoindentation) into the material using MTS Nanoindenter XP in continuous stiffness mode^42^ with the maximum load of 200 mN, each test was performed on nine testing position to avoid data deviation, and the penetration depth of every indentation was set at 1000 nm. Meanwhile, the accurate hardness was guaranteed by standardizing the fused silica standard sample. And the impression overviews were acquired by SEM.
